# Modified classification of surgical meshes for hernia repair based on the analyses of 1,000 explanted meshes

**DOI:** 10.1007/s10029-012-0913-6

**Published:** 2012-05-05

**Authors:** U. Klinge, B. Klosterhalfen

**Affiliations:** 1 Surgical Department, University Hospital of the RWTH Aachen, Pauwelsstraße 30, 52074 Aachen, Germany; 2 Institute for Pathology, Düren Hospital, Roonstr. 30, 52351 Düren, Germany

**Keywords:** Mesh classification, Porosity, Biomechanical anisotropy

## Abstract

**Background:**

It is an undisputable fact that meshes have become standard for repair of abdominal wall hernias. Whereas in the late eighties there were only a couple of different devices available, today we have to choose among some hundreds, with lots of minor and major variations in polymer and structure. As most of the minor variations may not lead to significant change in clinical outcome and may be regarded as less relevant, we should focus on major differences. Eventually, this is used to structure the world of mesh by forming groups of textile devices with distinct biological response. Many experimental and some clinical studies have underlined the outstanding importance of porosity, which fortunately, in contrast to other biomechanical quanlities, is widely unaffected by the anisotropy of meshes.

**Methods:**

In accordance with the major manufacturers of meshes, a classification of meshes was derived from a huge pool of textile data based briefly on the following: (1) large pores, (2) small pores, (3) additional features, (4) no pores, (5) 3D structure and (6) biological origin. At 1,000 explanted meshes the value of this classification was evaluated by group-specific assessment of inflammatory and connective tissue reaction.

**Results:**

Application of this classification to common products has proved feasable, and each of the six different classes includes devices that in clinical trials failed to show relevant differences in patients’ outcome when comparing products within the same group. Furthermore, histological analysis confirmed significant differences in tissue reactions between but not within the different classes.

**Conclusions:**

Classifying implants according to a similar response enables grouping patients into comparable cohorts despite implantation of different devices. Furthermore, it enables the examination of the impact of mesh classes for the various indications even from heterogenous data of registries. Finally and not the least, any grouping supports the surgeon to select the best device to meet the individual need and to tailor patients therapy.

**Electronic supplementary material:**

The online version of this article (doi:10.1007/s10029-012-0913-6) contains supplementary material, which is available to authorized users.

## Introduction

It was in the first edition of Hernia in 1997 where Amid [1] presented his extraordinary manuscript on the classification of biomaterials and its relation to complications in abdominal wall surgery. At that time, there were only a couple of different devices available, which were simply characterised mainly by the polymer used, without any further detail information. However, Amid already identified the porosity of meshes to be decisive for biocompatibility and their side effects. He defined 4 groups:

I Macroporous >75 μm
II Macro- with microporous
III Microporous
IV Submicronic pores/sheets.

Today more than 166 different devices are on the market [2], making it rather difficult for the surgeon to select the material best appropriate for his purpose, to test the impact of mesh material on outcome in clinical trials or to monitor the side effects of mesh materials in the present or upcoming registries. As most of the meshes at the time showed pores far larger than 75 μm, a revised classification was necessary.

In the late nineties, several experimental studies indicated that large pore and low-weight structures were found to be favourable [3, 4]. Later on because of difficulties in measuring porosity, this concept was shortened to the term “light-weight” concept reflecting a reduction of material. Recently in this journal Coda et al. [2] proposed a system involving the grouping of simple, composite or combined meshes, based on defining the weight;

1. Ultralight ≤35 g/m^2^
2. Light C 35–70 g/m^2^
3. Standard C 70–140 g/m^2^
4. Heavy C ≥140 g/m^2^.

However, it is not clear how to give a rational explanation for these weight borders. Though light weight usually indicates reduced material, it does not differentiate between film, fleece or net-like structure. Correspondingly, in 2006, Weyhe et al. [5] published inferior biocompatibility of a light-weight fleece structure without any larger pores, stressing the limited value of weight for the prediction of biocompatibility. In spite of the fact that new polymers are on the market with high specific weight resulting in heavy-weight meshes, their big pore size results in excellent biocompatibility [6, 7]. Other meshes are constructed as composites providing special features, which is as well not grasped by simple weight. Thus, Deeken et al. [8] separated meshes with additional barrier function used for intra-abdominal onlay plastic as a separate group.

Any characterisation of meshes by their biomechanical stability or elasticity, which may be considered as first choice because it is related to the task of meshes to reinforce the tissue, is limited by the fact that most of the meshes show a marked anisotropy[9–11]. This hinders the application of all uniaxial tests [12] and makes reliable comparisons impossible.

To overcome the confusion, we contacted major manufacturers of meshes in Germany and collected their physicochemical data of the products aiming to derive a system for grouping of meshes that is related to biocompatibility and helps the surgeon to keep an overview.

## Materials and methods

We got textile data of 55 different mesh devices for hernia repair from 9 manufacturers (Atrium, Bard, Braun-Aesculap, Covidien, Dynamesh, Ethicon, Gore, PFM, and Serag-Wiessner) organised in BVMED, Berlin, Germany (www.bvmed.de). As every company had specific and unique ways to characterise their product, we got a heterogeneous pattern of data comprising more than 40 possible variables from different tests. From this data pool, we derived rules for grouping of mesh devices, which were based on porosity as one of the measurements being robust against anisotropic effects. Eventually, this classification was evaluated by the manufacturers, who finally agreed to offer this proposal to the hernia societies for further discussion.

To evaluate whether the resulting grouping reflected different tissue response, we classified 1,000 explanted mesh samples, which have been sent to the institute for pathology, Düren in the years 2000–2010 according to this proposed classification, and analysed them for the reason of explantation (recurrence, pain or infection as indicated by the sending surgeon) and the intensity of inflammation and fibrosis around the mesh filaments.

Specimens were studied by light microscopy (LM). For LM, tissue samples were fixed in 10 % formalin, embedded in paraffin, and sections were stained with haematoxylin and eosin (H&E) and Elastica van Gieson (EvG). The morphometric evaluation consisted of a quantitative cell analysis of the inflammatory reaction and the soft-tissue reaction. Partial volumes (PV) of tissues were counted in 10 fields of 5 HE slides at a grid of 10 points (100×; area 0.1 mm^2^) within the interface of 0–300 μm. Parameters measured were the percentage share of the area covered by an inflammatory infiltrate (partial volume (PV) %) or by connective tissue (PV %).

Statistical analysis was carried out with SPSS 18.0 with ANOVA and Bonferroni post hoc test. A *p* < 0.05 was considered as significant.

## Results

The group of mesh devices offered by these nine manufacturers was rather inhomogeneous. It included 2D and 3D structures, flat meshes and plugs, monofilaments and multifilaments, absorbable or non-absorbable, with pores or without pores, with barrier or surface coating, as well as combinations of these. Even meshes with similar structures showed differences in stretch ability, anisotropy, hydrophilicity or structure stability under strain. As some tests were done only for few devices usually without providing the complete protocol for testing, we just present an extraction of data, which were available for at least most of the meshes.

**Fig. 1:**
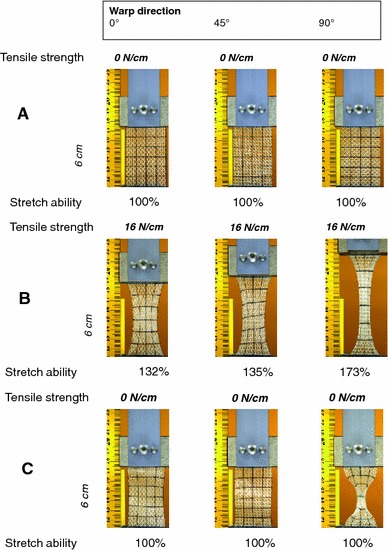
Grab test at a textile mesh structure that never has been implanted, to illustrate the difficulty to characterise stability and elasticity by uniaxial measurements **a** without strain, **b** at a strain of 16 N, **c** complete release (images with courtesy of FEG Textiltechnik, Aachen)

Measurements for weight ranged between 11 and 130 g/m^2^ with a variation of factor greater than 10. Assessment of stability in a uniaxial grab test resulted in a maximum tensile strength of 12–130 N/cm performed with strain in vertical direction (factor >10) and of 4–130 N/cm with strain in horizontal direction (factor of >30). Differences in the ratio of forces in a grab test done in two right-angled directions indicated the anisotropy of a mesh and ranges between 1 and 7. In the case of anisotropic mesh structures, tensile strength or elasticity was obviously influenced by the orientation of the mesh fibres, so that these parameters were inappropriate to specify the general characteristic of a mesh (Fig. 1). For some devices a two-dimensional ball pressing test was performed, however did not give comparable results because of different experimental conditions.

The force tearing out a seam showed a range from 9 to >60 N if strain was applied in vertical direction (factor of >6), or 8–>60 N in horizontal direction (factor of >6). The subsequent tearing force as indicator of firm bindings ranged from 4 to 212 N in vertical orientation and 4–160 N in horizontal orientation (factor of >40). However, these were measurements provided by the companies. The definition whether vertical or horizontal applications have been used obviously might not always reflect the main course of the filaments (warp direction) but seemed to be rather artificial and perhaps just a consequence of the packing process. Nevertheless, the big differences for many meshes between vertical and horizontal strain confirmed the considerable anisotropy of some devices questioning any uniaxial characterisation.

**Fig. 2:**
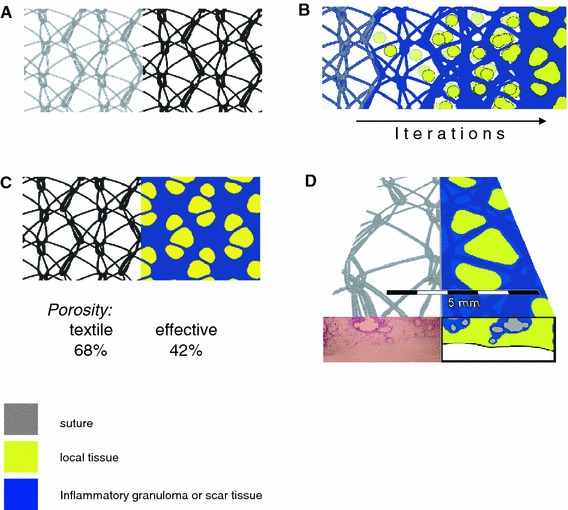
Textile or effective porosity of surgical meshes and the extent of bridging after tissue incorporation **a** textile class I construction with large pores (*left*), where the textile porosity (*right*) reflects in a black/white image all the area that is not covered by the filaments as percentage of the entire mesh area, **b** calculation of effective porosity according to Mühl et al. [13] has to consider that polypropylene meshes need a circular interfilament distance of ≥1,000 μm [20] to avoid bridging. Identification of “good” pores (*yellow*) is done by iterative fitting of spheres with a diameter of 1,000 μm into the area, which is not covered by either the filaments or its foreign body granuloma. The resulting area as percentage of the entire mesh area reflects the effective porosity. **c** Large pore mesh with a textile porosity of 68 % and an effective porosity of 42 %. **d** Large pore mesh with “good” pores that does not induce bridging (HE staining) but recovered by filling the pores with local fat tissue only consideration of the pores geometry allows to identify small pore meshes despite high textile porosity and low weight, which showed more inflammation than a heavy-weight construction with bigger pores (Weyhe et al. [5])

Porosity mainly is measured as the percentage of the area of the mesh, which is not covered by filaments, and then reflecting the textile porosity (Fig. 2), whereas the *effective* porosity represents only the area of “good” pores where bridging of scar tissue is avoided by sufficient interfilamentary distance as defined by Mühl et al. [13]. Rarely both types of information were provided by the manufacturers. Most often this *textile* porosity was determined as the area not covered by the mesh fibres, and the data varied between 0 and 97 %.

The mere textile data mostly assessed by standard textile tests appeared to be inappropriate for any grouping or comparison except for porosity. Thus, in consideration of (1) the outstanding importance of porosity and (2) the unaffectedness of porosity to anisotropy, we grouped the meshes into the following classes:

### Class I: Large pore meshes (characterised by a textile porosity of >60 % or an effective porosity of >0 %)

Though the relevance was not clear yet, we further subgrouped for

- Monofilament
- Multifilament
- Mixed structure or polymer (e.g. absorbable + non-absorbable, or different non-absorbable).

Actually, there is no clinical evidence that one type of filament is superior to another. Nevertheless, as many surgeons suspect an increased infection rate with multifilaments, the additional consideration of the filament type may be helpful for further analysis.

### Class II: Small pore meshes (characterised by a textile porosity of <60 % and without any effective porosity)

- Monofilament
- Multifilament
- Mixed structure or polymer.

### Class III: Meshes with special features

This group includes porous meshes with special features, for example, to prevent adhesions as realised in meshes with barrier function for intraperitoneal use or with surface coating, which were all difficult to compare with other devices.

### Class IV: Meshes with films

Because of the different biological integration film-like meshes without porosity, submicronic pore size or secondarily excised pores formed a specific group.

### Class V: 3D meshes

As all the pre-shaped, pre-formed, or 3D devices are rather difficult to characterise by simple biomechanic tests, all these devices are separated from the flat meshes in an individual group.

### Class VI: Biologicals

Eventually, the group for the so-called *Biologicals* complete the classification, though it has to be clarified whether absorbable/non-absorbable devices and biological/synthetic source may be differentiated. To start with, they may be subgrouped as

- Non-cross-linked
- Cross-linked
- Special features.

### Evaluation at 1,000 explanted mesh samples

For evaluating whether this classification has the potential to reflect a distinct biological response, we grouped 1,000 explanted mesh samples accordingly. We could identify 268 large pore class I meshes (brand names: Vypro, Ultrapro, Ti-mesh and Mersilene), 517 small pore class II devices (brand names: Marlex, Prolene, Atrium, Surgipro), 58 permanent films of class IV (ePTFE) and 157 plugs as devices for class V (Table 1).

**Table 1:** Explanted mesh samples and assignment to mesh class

Band name	Weight (g/m^2^)	Textile porosity	Class
Vypro	38	77	1
Ultrapro	28	67	1
Ti-mesh	35	68	1
Mersilene	40	71	1
Marlex	95	37	2
Prolene	109	56	2
Atrium	90	50	2
Surgipro	87	65	2*
ePTFE	400	0	4

Textile porosity reflects in a two-dimensional image the area that is not covered by the filaments; measurements were provided by the manufacture

* Meshes for class I were defined as structures with a textile porosity of at least 60 %. However, both monofilament and multifilament Surgipro meshes showed rather small pores, and as we microscopically could never see interfilament distances of more than 500 μm at explanted Surgipro meshes, we considered this mesh in accordance with Bellon et al. [24], Kapischke et al. [19] as small pore construction, though information provided by the manufacturers indicated a textile porosity of 65 %

Comparison of the indication for surgical revision revealed a significant impact of mesh class for all three different indications (chi-square *p* for infection *p* < 0.05; for pain or recurrence, *p* < 0.001; Table 2). In the groups of class II and V explants, infection and pain were significantly more often reported as reason for explantation than would have been expected in relation to the total share of these classes. In contrast, for class I meshes, the major indication for explantation was recurrence, whereas pain was significantly less often mentioned.

**Table 2:** Extraction of 1,000 explanted mesh samples and the reason for revision from the years 2000–2010 sent to the Institute for Pathology, Düren

	Infection	Pain	Recurrence
Class I (*n* = 268; 26.8 %)	37 (19 %)	30 (11 %)	210 (31 %)
Class II (*n* = 517; 51.7 %)	110 (56 %)	174 (65 %)	322 (48 %)
Class IV (*n* = 58, 5.8 %)	14 (7 %)	12 (5 %)	31 (5 %)
Class V (*n* = 157, 15.7 %)	37 (19 %)	51 (19 %)	108 (16 %)
Total	198 (100 %)	267 (100 %)	671 (100 %)

Some cases with more than one indication are reported

**Fig. 3:**
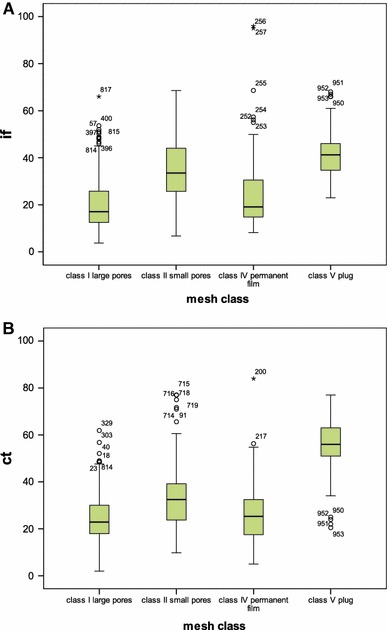
**a** Volume of inflammatory cells (*IF*), **b** volume of connective tissue (*CT*) at the interface of meshes in dependency of assigned mesh class. Outliers and extremes are depicted if >1.5 interquartile range above the 75th percentile. Analysing all meshes together the inflammatory volume differs significantly between the four classes (Bonferroni post hoc comparison with *p* < 0.01), with highest values for class V and class II but lowest for class I. The volume of fibrosis differs significantly between the four classes (Bonferroni post hoc comparison with *p* < 0.01), with the class I (*p* = 0.765), again with highest values for class V and class II but lowest for class I. The interfering impact of the indication for mesh removal manifests mainly at the extremes. Regarding IF from samples with values above the 95 % quartile, 12/12 class I meshes, 8/24 class II, 2/2 class IV, and 6/7 class V have been explanted because of infection. Regarding CT from samples with values above the 95 % quartile most often the explantation was done because of recurrence 11/13 of class I, 20/25 of class II, 1/2 of class IV and 5/7 of class V

Quantifying the amount of inflammatory and connective tissue in the mesh area confirmed significant differences between the mesh classes with highest values for small pore meshes or plugs (Fig. 3; Table 3).

**Table 3:** Volume of inflammatory cells (*IF*) or connective tissue (*CT*) of 1,000 mesh samples, explanted for pain, infection or recurrence

	*N*	Median	Interquartile range	Range
*IF*				
Class I large pores	268	17.1	13.4	62.3
Class II small pores	517	33.5	18.4	61.9
Class IV permanent film	58	19.1	16.8	87.8
Class V plug	157	41.2	11.7	45.0
Total	1,000	30.6	21.9	92.3
*CT*				
Class I large pores	268	22.9	12.1	59.9
Class II small pores	517	32.5	15.4	67.2
Class IV permanent film	58	25.3	15.8	79.0
Class V plug	157	56.0	12.5	56.5
Total	1,000	31.2	20.1	82.0

When using all specimens regardless the different indications for explantation all possible comparisons between mesh classes showed significant differences with *p* < 0.01, with only one exception for CT when comparing class I with class IV (ANOVA with Bonferroni, *p* = 0.765)

Repetition of the analysis separately for the indication recurrence, pain or infection similarly revealed significant differences, except for

Recurrence: IF between class I and class IV; CT between class II and class IV

Chronic pain: IF between class I and class IV; CT between class I and class II and IV

Infection: IF between class V and classes I, II and IV, between class I and class II; CT between class I and class IV

## Discussion

Any attempt to implement a classification of meshes for hernia surgery has to explain its right to exist. The most important argument in this regard is that we need it for our own quality control, to learn whether some devices may be related with more adversal events than others. Concerning repeated reports on medical devices causing serious problems, there is an ongoing discussion about new regulations for the approval of medical devices [14] stressing the necessity for an intensified post-market surveillance. As the incidences of mesh-related complications in the field of hernia surgery are expected to be far less than 5 % per year, any evaluation by clinical trials is hardly feasible with sufficient statistical power and thus will likely end up in insignificant results [15]. One alternative may be an obligate complete follow-up by unique device identifiers, but this will mean tremendous paper work for all of us and whether we get sufficient information is questionable.

The best known alternative for quality control of medical devices is data from comprehensive registries, as it already exists in some countries and is prepared as EuraHS (www.eurahs.eu), an international Internet-based platform [16]. There is no doubt that any documentation in a registry of hernia surgery has to include the type of mesh. The question is “how to do it”? Considering the many different brand names of even similar products, any evaluation will be rather difficult, particularly with respect to having >200 devices with >300 trade names and perhaps 50 new developments each year, maybe except for some devices of large international manufacturers. Latest at that point we should think about a way to bring all the different devices together in some few groups of distinct biological reaction.

Any grouping should focus on relevant “major” differences. Among the many textile characteristics, the only property that can be used today for grouping is the porosity. There is some evidence that large pore flat structures are beneficial with less complaints in comparison with small pore structures [17, 18]. Unfortunately, we do not have sufficient parameters to characterise other devices as plugs or meshes with 3D deformation, which therefore have to be collected in a separate group, as well as all the meshes with additional features, for example, with barrier function for the use in the abdominal cavity or with supplementary surface coating to direct the local wound healing. Not neglecting the fact that devices without pores have to be considered as a separate group because of the fibrotic capsule formation. Whether films with artificial perforation should be considered as film or mesh is not clear.

For most meshes there is a close relation between weight and porosity. But there are exceptions showing that grouping according to weight as well as classification by porosity is imperfect and disputable. We chose a textile porosity of 60 % to classify between class I and class II as from our textile data this value seems to be the appropriate border. However, Kapischke et al. [19] measured a porosity of <40 % in the case of small pore meshes and of >50 % (but <60 %) in the case of large pore meshes. However, as for the weight any definition of large or small pore is somehow arbitrary and is influenced by the test conditions.

Furthermore, an interfilament distance of 1,000 μm was used for the calculation of the effective porosity (Fig. 2), because for polypropylene meshes this is the least distance that prevents bridging of scar tissue, which then fills out the entire pore [20]. In the microscopic examination we usually observed interfilament distances of more than 500 μm in sections of explanted class I meshes, whereas this could never be seen in class II meshes. However, it still is open for further studies whether 500 μm is a reliable limit for histology and 1,000 μm for the calculation of the effective porosity or whether this should be modified. Though a standardised measurement of the effective porosity that considers only large pores with sufficient geometry to avoid bridging may best provide a qualitative criterion to differentiate these two groups [13], it may be speculated whether the assumption of a best pore size of 1,000 μm for preserving an effective porosity has to be adjusted for; for example, meshes of polyvinylidenfluoride PVDF with its smaller foreign body granuloma do not show bridging of scar throughout the pores even at small pore sizes of less than 650 μm whereas polypropylene monofilaments usually do [20, 21].

Because of the rapid degradation of the absorbable parts of composite meshes, this grouping of meshes is only based on the properties of the permanent parts. Only if a persistent effect on the local tissue response is assumed, these meshes should be classified as class III, for example, as improving local healing with bioactive supplements.

In 1997, Amid specified meshes with tiny pores of less than 75 μm as these are suspected to favour infections. Recently, however, controversial discussion rather focuses on light weight versus heavy weight or large pore versus small pore, respectively. Therefore, we decided not to keep this subgrouping with micropore devices.

The meshes of class III mainly are not supposed to be comparable with class I and class II meshes, because of their additional features. As most of the IPOM meshes include some films as temporary barrier, its porosity seems to be difficult to define, in particular, as it is the barrier rather than the porosity of the textile construction, which determines ingrowth and adhesion formation. Correspondingly, all the IPOM meshes were put into this group. As there are not so many, this seems to be an acceptable compromise at the moment.

In the present classification, class V includes a huge variety of different 3D devices, which in common are difficult to characterise. We still do not have sufficient skills to measure the properties and characteristics of 3D devices. We are still limited to show that large pore constructions in a 3D device behave differently from small pore 3D devices.

Class VIc should include absorbable materials that cannot be sufficiently described by the presence of cross-linkage, for example, the synthetic absorbable materials, synthetic collagens, combinations of textile structures with cells for tissue engineering. However, while it might not be necessary to have this subgroup today, it most likely will be filled up in future.

The analysis of the explanted meshes clearly demonstrated the distinct tissue response between the mesh classes. Considering the 3D architecture of plugs it is no surprise to find the biggest amount of inflammation and of connective tissue in their dense network of alloplastic fibres. This is followed by the small pore meshes of class II, which is in accordance with several experimental data [22], whereas PTFE films and the large pore structures of class I showed less intense inflammatory response. As ePTFE is often used within the abdominal cavity, this low intensity of inflammation and fibrosis may reflect the loose intra-abdominal adhesions rather than the fibrotic capsule around the mesh.

Infection and pain as indication for mesh removal were more often seen with meshes of class II (56 and 65 %, respectively), significantly more than should be expected if infection and pain are unaffected by the mesh class and only reflect the percentage among the 1,000 explants (52 %). Recurrence as reason for revision was predominantly reported with devices of class I. This relatively high rate of recurrences as indication for mesh removal may be caused by the fact that infection or pain as indication for mesh removal is less often seen, which consecutively increases the percentage of recurrence. An increased recurrence rate by the use of light-weight meshes has been checked in clinical studies and could widely be excluded, at least for groin hernia [17, 23]. These data do not reflect the absolute risk of a specific device for infection, pain or recurrence, because we do not know the number of implanted mesh materials. However, any major deviation from the normal distribution should capture our attention, and as a consequence to exclude any mesh-related problem, we should start subsequent studies.

The present grouping is based on the assumption that meshes within a group are widely comparable or show only minor differences, and minor differences are defined by the impossibility to prove any relevant differences in a RCT. In collaboration with manufacturers organised in the German Medical Technology Association, it is planed to provide this classification on the webpages of the hernia societies, performing regular updates and adding new devices, once essential information of the product such as porosity is provided. Future challenge is the definition of standards for mesh characterisation, particularly in the case of anisotropic properties, to find commonly accepted standards for measuring (effective) porosity and to specify the limits, which indicate the “good pores” or a distinct tissue integration, not least allowing objective confirmation of the information provided by the manufacturer. Perhaps hydrophilicity, diameter of filaments or something new currently not known will eventually be added do identify superior medical devices. The past collaboration in this project clearly showed that surgeons and manufacturer are indeed willing to work together for the benefit of the patient.

However, this proposal of a classification cannot fulfil all demands or requests to place a certain device as unique or at the top of a group. As it should provide a rationale to help surgeons to get an overview on important material properties of hundreds of different devices, this proposal operates with generalisations bringing different and incomparable data into a clear scheme. Basically, the definition of a specific group should summarise all devices among which a significantly different impact on outcome could not be demonstrated in a clinical trial. Similarly any significant differences in outcome in clinical trials should result in distinct groups. During numerous intense discussions with the engineers of the manufacturing companies, we could not identify any more promising alternatives, and thus manufacturers have agreed to adopt this classification, which of course has to be evaluated with the data of upcoming studies or registries, and will likely be modified according to our improved knowledge and the demands of the surgeons.

The aim of this classification is to group the many different meshes so that the impact of material can now be evaluated systematically from the data of studies and registries. Today there is no chance to make any reliable analysis with focus on the material, as the current recording of mesh material is restricted to brand names. This new grouping that puts together comparable mesh materials will help to evaluate the impact of material principals on the outcome. A first attempt to evaluate the separation of class I and class II was done in this manuscript by histological analysis of explanted mesh samples. In fact, we could demonstrate that class I and class II meshes are related to significant differences in tissue response, whereas the differences within a class are considerable lower. It becomes obvious that the definitions of this classification fulfil the purpose of classifying distinct tissue integrations.

## Electronic supplementary material

Below is the link to the electronic supplementary material.

Supplementary material 1 (PDF 62 kb)
