# Supplementary material for: Modified classification of surgical meshes for hernia repair based on the analyses of 1,000 explanted meshes
Source: Hernia. 2012 May 5;16(3):251–8. doi: 10.1007/s10029-012-0913-6 (PMC3360857; doi:10.1007/s10029-012-0913-6)
Supplement: Supplementary file 1 — Supplementary material 1 (PDF 62 kb) [file 10029_2012_913_MOESM1_ESM.pdf]

Synthetic mesh

Application	Detailed Application	Company´s	Group 1<br>Advanced technology<br>„large pores“	Group 2<br>Basic technology<br>„small pores“	Group 3,4<br>Special features, and films<br>„prevention of adhesion, modified scar formation, bioactive mesh, drug release etc.“	Group 5<br>preshaped, preformed, 3D<br>„for in situ regeneration“
Groin	Inguinalis	**Atrium**		Prolite & Prolite Ultra Mesh	C-Qur FX Filament Coated, Pro Loop Plug	
		**B. Braun**	Optilene	Premilene		
		**C.R.Bard**	Bard SoftMesh	Bard Flatmesh, Bard Preshape	Bard Perix Plug, Bard Perfix Light Plug, Polysoft	Bard 3D Max, Bard 3D Max Light
		**Cook**				
		**Covidien**	Parietene™ - Parietene™ Light - Parietex™ Type C - Parietex™ type T - Parietex™ Mono	Surgpiro™	Parietene™ Progrip - Parietex™ Progrip	Surgipro™ Hernia Mate Plug - Parietex™ Progrip PlugNpatch - Parietex™ Presaped & folding meshes - Parietex™ Progrip Anatomic
		**DynaMesh**	DynaMesh Lichtenstein, DynaMesh Endolap, DynaMesh-PP Light, DynaMesh-PP Standard			DynaMesh Lichtenstein
		**Ethicon**	Mersilene	Prolene	Ultrapro, UPP, UHS, Proceed, Physiomesh, VYPRO 11	**PHS, UHS, UPP, 3DP**
		**Gore**		Infinite		
		**KCI**				
		**PFM medcial**	TiMESH extralight, light, strong / TiPATCH / TiPLUG System / TiLENE Plug / TiLENE Plug Set		TiMESH extralight, light, strong / TiPATCH / TiPLUG System / TiLENE Plug / TiLENE Plug Set	
		**Serag Wiesener**	SeraMesh XO	SeraMesh SO		
		**Tutogen**				
	Femoralis	**Atrium**		Prolite & Prolite Ultra Mesh	C-Qur FX Filament Coated, Pro Loop Plug	
		**B. Braun**	Optilene	Premilene		
		**C.R.Bard**	Bard SoftMesh	Bard Flatmesh, Bard Preshape	Bard Perix Plug, Bard Perfix Light Plug, Polysoft	Bard 3D Max, Bard 3D Max Light
		**Cook**				
		**Covidien**	Parietene™ - Parietene™ Light - Parietex™ Type C - Parietex™ type T - Parietex™ Mono	Surgpiro™	Parietene™ Progrip - Parietex™ Progrip	Surgipro™ Hernia Mate Plug - Parietex™ Progrip PlugNpatch - Parietex™ Presaped & folding meshes
		**DynaMesh**	DynaMesh Endolap, DynaMesh-PP Light, DynaMesh-PP Standard			
		**Ethicon**	Mersilene	Prolene	Ultrapro, UPP, UHS, Proceed, Physiomesh, VYPRO 11	**PHS, UHS, UPP, 3DP**
		**Gore**		Infinite		
		**KCI**				
		**PFM medcial**	TiMESH extralight, light, strong / TiPATCH / TiPLUG System / TiLENE Plug / TiLENE Plug Set		TiMESH extralight, light, strong / TiPATCH / TiPLUG System / TiLENE Plug / TiLENE Plug Set	
		**Serag Wiesener**	SeraMesh XO	SeraMesh SO		
		**Tutogen**				
Ventral	Parastomal	**Atrium**		ATRIUM Prolite&Prolite Ultra Mesh	ATRIUM C-Qur FX Filament coated, C-Qur	
		**B. Braun**	Optilene	Premilene	Omyra	
		**C.R.Bard**			Bard Parastomal Patch	
		**Cook**				
		**Covidien**	Parietene™ - Parietene™ Light - Parietex™ Type C - Parietex™ type T - Parietex™ Mono	Surgpiro™	Parietex™ Composite Parastomal	
		**DynaMesh**	DynaMesh-PP Light, DynaMesh-PP Standard			DynaMesh-IPST
		**Ethicon**	Mersilene	Prolene	Ultrapro, Proceed, Physiomesh, VYPRO, VYPRO 11	
		**Gore**				
		**KCI**				
		**PFM medcial**	TiMESH light , strong / TiLENE Guard / TiLENE Guard Set		TiMESH light , strong / TiLENE Guard / TiLENE Guard Set	
		**Serag Wiesener**				
		**Tutogen**				
	Umbilicalis	**Atrium**		ATRIUM Prolite&Prolite Ultra Mesh	ATRIUM C-Qur FX Filament Coated, C-Qure V-Patch, C-Qur	
		**B. Braun**	Optilene	Premilene	Omyra	
		**C.R.Bard**			Bard Ventralex	
		**Cook**				
		**Covidien**	Parietene™ - Parietene™ Light - Parietex™ Type C - Parietex™ type T - Parietex™ Mono	Surgpiro™	Parietene™ Progrip - Parietex™ Progrip	Parietex™ Umbilical patch
		**DynaMesh**	DynaMesh-CICAT, DynaMesh-PP Light, DynaMesh-PP Standard		DynaMesh-IPOM	
		**Ethicon**	Mersilene	Prolene	Ultrapro, Proceed, Physiomesh, VYPRO,VYPRO 11	**PVP, UHS**
		**Gore**			DualMesh	
		**KCI**				
		**PFM medcial**	TiMESH light, strong / TiLENE Plug / TiLENE Plug Set		TiMESH light, strong / TiLENE Plug / TiLENE Plug Set	
		**Serag Wiesener**				
		**Tutogen**				
	Epigastrica	**Atrium**		ATRIUM Prolite&Prolite Ultra Mesh	ATRIUM C-Qur FX Filament Coated	
		**B. Braun**	Optilene	Premilene		
		**C.R.Bard**				
		**Cook**				
		**Covidien**	Parietene™ - Parietene™ Light - Parietex™ Type C - Parietex™ type T - Parietex™ Mono	Surgpiro™	Parietene™ Progrip - Parietex™ Progrip	
		**DynaMesh**	DynaMesh-CICAT, DynaMesh-PP Light, DynaMesh-PP Standard			
		**Ethicon**	Mersilene	Prolene	Ultrapro, Proceed, Physiomesh, VYPRO, VYPRO 11	**PVP, UHS**
		**Gore**				
		**KCI**				
		**PFM medcial**	TiMESH light, strong		TiMESH light, strong	
		**Serag Wiesener**				
		**Tutogen**				
	Diaphragmatica	**Atrium**		ATRIUM Prolite&Prolite Ultra Mesh	ATRIUM C-Qur FX Filament Coated, C-Qur	
		**B. Braun**			Omyra	
		**C.R.Bard**			Bard Crurasoft Patch	
		**Covidien**	Parietene™ Composite - Parietex™ Composite		Parietene™ Composite - Parietex™ Composite	
		**DynaMesh**				
		**Ethicon**	Mersilene	Prolene	Ultrapro, Proceed, Physiomesh, VYPRO, VYPRO 11	**PVP, UHS**
		**Gore**				
		**KCI**				
		**PFM medcial**	TiMESH light, strong / TiSURE		TiMESH light, strong / TiSURE	
		**Serag Wiesener**				
		**Tutogen**				
	Narbenhernie	**Atrium**		ATRIUM Prolite&Prolite Ultra Mesh	ATRIUM C-Qur, ATRIUM C-Qure TacShield, V-Patch	
		**B. Braun**	Optilene	Premilene	Omyra	
		**C.R.Bard**	Bard SoftMesh	Bard Flatmesh	Bard Ventrio, Bard Composix L/P, Bard Composix Kugel, Bard	
		**Covidien**	Parietene™ Light X-Shaped - Parietene™ - Parietene™ Light - Parietex™ Type C - Parietex™ type T - Parietex™ Mono	Surgpiro™	Parietene™ Progrip - Parietex™ Progrip	
		**DynaMesh**	DynaMesh-CICAT, DynaMesh-PP Light, DynaMesh-PP Standard			
		**Ethicon**	Mersilene	Prolene	Ultrapro, Proceed, Physiomesh, VYPRO, VYPRO 11	**PVP, UHS**
		**Gore**				
		**KCI**				
		**PFM medcial**	TiMESH light, strong / TiLENE Strip		TiMESH light, strong / TiLENE Strip	
		**Serag Wiesener**				
		**Tutogen**				
	Abdominal	**Atrium**		ATRIUM Prolite&Prolite Ultra Mesh	ATRIUM C-Qur FX Filament Coated, C-QUR TacShield, C-Qur	
		**B. Braun**	Optilene	Premilene	Omyra	
		**C.R.Bard**	Bard SoftMesh	Bard Flatmesh	Bard Ventrio, Bard Composix L/P, Bard Composix Kugel, Bard	
		**Cook**				
		**Covidien**	Parietene™ Light X-Shaped - Parietene™ - Parietene™ Light - Parietex™ Type C - Parietex™ type T - Parietex™ Mono	Surgpiro™	Parietene™ Progrip - Parietex™ Progrip	
		**DynaMesh**	DynaMesh-CICAT, DynaMesh-PP Light, DynaMesh-PP Standard			
		**Ethicon**	Mersilene	Prolene	Ultrapro, Proceed, Physiomesh, VYPRO, VYPRO 11	**PVP, UHS**
		**Gore**				
		**KCI**				
		**PFM medcial**	TiMESH light, strong / TiLENE Strip		TiMESH light, strong / TiLENE Strip	
		**Serag Wiesener**				
		**Tutogen**				
	kongenitale Bauchwanddefekte (Omphalozele, Laparoschisis)	**Atrium**		ATRIUM Prolite&Prolite Ultra Mesh	ATRIUM C-Qur FX Filament Coated, C-Qur, TacShield	
		**B. Braun**				
		**C.R.Bard**				
		**Cook**				
		**Covidien**				
		**DynaMesh**				
		**Ethicon**			PHYSIOMESH, PROCEED	PVP
		**Gore**				
		**KCI**				
		**PFM medcial**				
		**Serag Wiesener**				
		**Tutogen**				
Intraperitoneal		**Atrium**			TacShield, C-Qur, V-Patch	
		**B. Braun**			Omyra	
		**C.R.Bard**			Bard Ventrio, Bard Composix L/P, Bard Composix Kugel, Bard Composix EX, Bard Sepramesh IP Composite, Bard Dulex	
		**Cook**				
		**Covidien**	Parietene™ Composite - Parietex™ Composite - Parietex™ Composite Open skirt		Parietene™ Composite - Parietex™ Composite - Parietex™ Composite Open skirt	
		**DynaMesh**			DynaMesh-IPOM	DynaMesh-IPST
		**Ethicon**				
		**Gore**				
		**KCI**				
		**PFM medcial**	TiMESH light, strong / TiPATCH / TiPLUG System / TiLENE Plug / TiLENE Plug Set / TiSURE / TiLENE Guard / TiLENE Guard Set / TiLENE Strip		TiMESH light, strong / TiPATCH / TiPLUG System / TiLENE Plug / TiLENE Plug Set / TiSURE / TiLENE Guard / TiLENE Guard Set / TiLENE Strip	
		**Serag Wiesener**				
		**Tutogen**				

Biological devices

Application	Detailed Application	Company´s	xenografts			allografts		
			crosslinked	non crosslinked	special features	crosslinked	non crosslinked	special features
Groin	Inguinalis	Atrium						
		B.Braun						
		C.R.Bard	Bard Collamend FM		Bard Collamend FM			
		Cook		BIODESIGN				
		Covidien	PERMACOL					
		DynaMesh						
		Ethicon						
		Gore						
		KCI		STRATTICE				
		PFM medcial						
		Serag Wiesener						
		Tutogen		Tutomesh				
	Femoralis	Atrium						
		B.Braun						
		C.R.Bard	Bard Collamend FM		Bard Collamend FM			
		Cook		BIODESIGN				
		Covidien						
		DynaMesh						
		Ethicon						
		Gore						
		KCI		STRATTICE				
		PFM medcial						
		Serag Wiesener						
		Tutogen		Tutomesh				
Ventral	Parastomal	Atrium						
		B.Braun						
		C.R.Bard	Bard Collamend FM		Bard Collamend FM			
		Cook		BIODESIGN				
		Covidien	PERMACOL					
		DynaMesh						
		Ethicon						
		Gore						
		KCI		STRATTICE				
		PFM medcial						
		Serag Wiesener						
		Tutogen		Tutomesh				
	Umbilicalis	Atrium						
		B.Braun						
		C.R.Bard	Bard Collamend FM		Bard Collamend FM			
		Cook		BIODESIGN				
		Covidien	PERMACOL					
		Ethicon						
		Gore						
		KCI		STRATTICE				
		PFM medcial						
		Serag Wiesener						
		Tutogen		Tutomesh				
	Epigastrica	Atrium						
		B.Braun						
		C.R.Bard	Bard Collamend FM		Bard Collamend FM			
		Cook		BIODESIGN				
		Covidien	PERMACOL					
		DynaMesh						
		Ethicon						
		Gore						
		KCI		STRATTICE				
		PFM medcial						
		Serag Wiesener						
		Tutogen		Tutomesh				
	Diaphragmatica	Atrium						
		B.Braun						
		C.R.Bard	Bard Collamend FM		Bard Collamend FM			
		Cook		BIODESIGN				
		Covidien	PERMACOL					
		DynaMesh						
		Ethicon						
		Gore						
		KCI		STRATTICE				
		PFM medcial						
		Serag Wiesener						
		Tutogen		Tutomesh				
	Narbenhernie	Atrium						
		B.Braun						
		C.R.Bard	Bard Collamend FM		Bard Collamend FM			
		Cook		BIODESIGN				
		Covidien	PERMACOL					
		DynaMesh						
		Ethicon						
		Gore						
		KCI		STRATTICE				
		PFM medcial						
		Serag Wiesener						
		Tutogen		Tutomesh				
	Abdominal	Atrium						
		B.Braun						
		C.R.Bard	Bard Collamend FM		Bard Collamend FM			
		Cook		BIODESIGN	mit VAC kombinierbar			
		Covidien	PERMACOL					
		DynaMesh						
		Ethicon						
		Gore						
		KCI		STRATTICE				
		PFM medcial						
		Serag Wiesener						
		Tutogen		Tutomesh	Tutogen Tutomesh in Kombination mit VAC möglich.			
	kongenitale Bauchwanddefekte (Omphalozele, Laparoschisis)	Atrium						
		B.Braun						
		C.R.Bard	Bard Collamend FM		Bard Collamend FM			
		Cook		BIODESIGN				
		Covidien	PERMACOL					
		DynaMesh						
		Ethicon						
		Gore						
		KCI		STRATTICE				
		PFM medcial						
		Serag Wiesener						
		Tutogen		Tutomesh				
Intraperitoneal		Atrium						
		B.Braun						
		C.R.Bard	Bard Collamend FM		Bard Collamend FM			
		Cook		BIODESIGN				
		Covidien	PERMACOL					
		DynaMesh						
		Ethicon						
		Gore						
		KCI		STRATTICE				
		PFM medcial						
		Serag Wiesener						
		Tutogen						
